# Electroconvulsive therapy and the WHO: patients and professionals deserve expertise and facts, not ideology

**DOI:** 10.1007/s00115-025-01891-x

**Published:** 2025-08-25

**Authors:** David Zilles-Wegner, Alexander Sartorius

**Affiliations:** 1 Klinik für Psychiatrie und Psychotherapie, Universitätsmedizin Göttingen, Georg-August-Universität, Von-Siebold-Str. 5, 37075 Göttingen, Germany; 2 Klinik für Psychiatrie und Psychotherapie, Zentralinstitut für Seelische Gesundheit, J 5, 68159 Mannheim, Germany

Recently, electroconvulsive therapy (ECT) has once again become the target of allegations that were thought to have been overcome. An official document from the World Health Organization (WHO; [13]) and a corresponding letter in *Lancet Psychiatry* [3] contain statements that are both stigmatizing and scientifically incorrect. These persistent prejudices must be clearly refuted to prevent potential harm to patients through the failure to provide the indicated treatment. In summary, the authors address the following aspects (original wording indicated by quotation marks):

1. ECT causes “brain damage” or at least “maladaptive” brain changes.
2. ECT is associated with “serious risks” (i.e., mortality, cardiovascular and persistent cognitive effects like “complete memory erasure”).
3. “Using ECT for children is not recommended and should be prohibited through legislation.”
4. ECT “must only be administered with the written or documented, free and informed consent of the person concerned.” “ECT without informed consent can constitute torture or ill-treatment.”

In the following, we will address these points individually and present the current scientific evidence.

1. The most current of three publications cited as “recent research” and that purportedly prove brain damage caused by ECT was published in 2014 [3]. Only one study actually showed a reduction in regional brain volume correlated with the number of ECT sessions, which the authors of the original study interpreted as an expression of depression severity. In contrast to this highly biased selection, there is robust evidence in the current neuroimaging literature that ECT is associated with (temporary) increases in gray matter volumes. Although these macroscopic changes are not consistently associated with symptom improvement, the underlying neuroplastic processes may be a necessary condition for clinical response [4]. Furthermore, studies investigating highly sensitive markers of neural damage, such as neurofilament light chain, clearly contradict any negative impact on brain structure [9]. At present, there is no evidence of any structural cerebral damage caused by ECT.
2. ECT is not associated with increased mortality or with permanent cognitive impairment. By contrast, several large-scale register studies show protective effects of ECT with regard to all-cause mortality and cardiovascular events in comparison with patient cohorts not treated with ECT [6]. Receiving ECT to treat depression is not associated with an increased risk of dementia [5]. The literature cited to support the notion of permanent cognitive impairment following ECT is exclusively qualitative and refers to selected and drastic statements made by individual patients [3]. When objectively measured by neuropsychological tests, cognitive side effects vanish after a few days to weeks, and patients ultimately show better cognitive performance on average compared to baseline [8]. Despite the fact that patients treated with ECT may experience reduced autobiographical memory consistency exceeding the level associated with severe depression per se, meta-analyses of quantitative studies involving several thousand patients show an average improvement in both objective and subjective cognitive performance [7, 8]. Furthermore, the improvement in depressive symptoms after ECT is associated with objective and subjective improvements in cognition [1, 7]. Therefore, the best way to improve cognitive functioning in patients with severe and debilitating psychiatric illness is still the effective treatment of symptoms, including the option of ECT.
3. Although ECT is more effective in older patients compared to adolescents in the context of depression and schizophrenia, there are specific indications in child psychiatry that ECT may represent the superior (and sometimes the only lifesaving) treatment option, e.g., severe and malignant catatonia, severe behavioral stereotypies, and self-harm in autism spectrum disorders [11]. The authors raise general “concerns about the adverse effects of applying electricity to developing brains” but do not substantiate these concerns in any way. According to them, they are “particularly vulnerable to trauma,” which wrongly portrays ECT as traumatic (see above [9]). The consequences of the alternative, i.e., not treating severely affected patients at all, are not discussed. From a clinical perspective, it is difficult to imagine that remaining in a state of severe psychosis, depression with suicidality, or catatonia is the healthier alternative for a developing brain. Withholding ECT in such cases is unethical and would constitute failure to provide assistance.
4. As the most severe forms of psychiatric illness may be associated with a lack of decision-making capacity, consent may be given by a legal substitute (depending on the local legislation). A meta-analysis has shown that lack of decision-making capacity is associated with an even better response to ECT [10], most likely due to the accumulation of positive predictors of ECT response (higher age, symptom severity, psychotic/psychomotor symptoms) in the affected patients. Patients initially treated under coercion have good overall response rates and identically high future approval rates for ECT compared to those who were treated with consent [12]. The German Federal Court of Justice (BGH) has ruled that, within the narrow framework of a fundamentally restrictive indication regime, compulsory ECT does not constitute a cruel, inhuman, or degrading treatment in violation of Article 15 of the UN Convention on the Rights of Persons with Disabilities. By contrast, in certain cases it may even be a fulfillment of the state’s duty to grant protection to people in need by providing them with medical care (BGH, XII ZB 191/21).

Regardless of these specific aspects, the ECT-related statements [13] are almost identical to those in a previous WHO publication [14]. This document mentions a member of the Citizens Commission on Human Rights (CCHR) under “External contributors.” According to the Bavarian State Office for the Protection of the Constitution (*Bayerisches Landesamt für Verfassungsschutz*), the CCHR is a front organization of Scientology, and psychiatrists and psychologists are the focus of CCHR’s targeted antipsychiatry campaigns. Against this backdrop, the stance of the letter becomes clearer. Nevertheless, the authors assure that the guidance has been “developed with the input of leading experts” and that the “safety, ethics, and effectiveness of ECT remain contested around the world and among top authorities in the field” [3]. At least with regard to ECT, we seriously question the expertise and experience of the contributing experts and authorities. Many studies show that greater personal qualification, formal training, and direct clinical experience with ECT are associated with more positive attitudes toward its use among healthcare professionals. Even more important, personal experience with ECT is associated with more positive attitudes among patients, caretakers, and relatives. Those who have experienced ECT on both a professional and personal level probably have the most nuanced view of it. And they are the ones who wrote some of the most impressive pleas in defense of ECT [2]: “Medicine’s calling is to heal, not to judge. Let us honor this by confronting stigma with truth, empathy, and unwavering resolve.”

## Practical conclusion

- Misleading and stigmatizing statements about electroconvulsive therapy (ECT) must be corrected.
- ECT is a very safe and mostly well-tolerated treatment that is associated with reduced overall mortality.
- Neuroplastic effects are presumably part of the mechanism of action; even highly sensitive methods have shown no evidence of neuronal damage.
- ECT is associated with improved objective and subjective cognitive performance, depending on the clinical response.
- ECT can restore the capacity to consent in severely affected patients, thereby promoting autonomy.

## Supplementary Information

Supplementary References
