# Supplementary material for: Electroconvulsive therapy and the WHO: patients and professionals deserve expertise and facts, not ideology
Source: Nervenarzt. 2025 Aug 25;97(4):397–9. doi: 10.1007/s00115-025-01891-x (PMC13314889; doi:10.1007/s00115-025-01891-x)
Supplement: Supplementary file 1 — Supplementary References [file 115_2025_1891_MOESM1_ESM.docx]

**Supplementary References**

1. Alcoverro-Fortuny Ò, Rueda SL, Esnaola M et al (2025) Stigma Regarding Electroconvulsive Therapy Among Healthcare Professionals. J ECT. https://doi.org/10.1097/YCT.0000000000001128

2. Anderson IM, McAllister-Williams RH, Downey D et al (2021) Cognitive function after electroconvulsive therapy for depression: relationship to clinical response. Psychol Med 51:1647–1656. https://doi.org/10.1017/S0033291720000379

3. Aoki Y, Yamaguchi S, Ando S et al (2016) The experience of electroconvulsive therapy and its impact on associated stigma: A meta-analysis. Int J Soc Psychiatry 62:708–718. https://doi.org/10.1177/0020764016675379

4. Barchas RE (2025) Electroconvulsive therapy highlights the urgency of addressing medical stigma. Lancet Reg Health Am 47:101154. https://doi.org/10.1016/j.lana.2025.101154

5. Besse M, Belz M, Bartels C et al (2024) The myth of brain damage: no change of neurofilament light chain during transient cognitive side-effects of ECT. Eur Arch Psychiatry Clin Neurosci 274:1187–1195. https://doi.org/10.1007/s00406-023-01686-8

6. Dwork AJ, Arango V, Underwood M et al (2004) Absence of histological lesions in primate models of ECT and magnetic seizure therapy. Am J Psychiatry 161:576–578. https://doi.org/10.1176/appi.ajp.161.3.576

7. Funk M, Drew Bold N, Francesca Moro M et al (2025) Guidance on mental health policy and strategic action plans: Module 2. Key reform areas, directives, strategies, and actions for mental health policy and strategic action plans. World Health Organization, Geneva

8. Funk M, Drew N, Pathare S et al (2025) Electroconvulsive therapy: reaffirming the case for caution, consent, and rights. The Lancet Psychiatry. https://doi.org/10.1016/S2215-0366(25)00192-0

9. Gbyl K, Videbech P (2018) Electroconvulsive therapy increases brain volume in major depression: a systematic review and meta-analysis. Acta Psychiatr Scand 138:180–195. https://doi.org/10.1111/acps.12884

10. Gergel T (2022) „Shock tactics“, ethics and fear: an academic and personal perspective on the case against electroconvulsive therapy. Br J Psychiatry 220:109–112. https://doi.org/10.1192/bjp.2021.116

11. Grover SK, Chakrabarti S, Khehra N, Rajagopal R (2011) Does the experience of electroconvulsive therapy improve awareness and perceptions of treatment among relatives of patients? J ECT 27:67–72. https://doi.org/10.1097/YCT.0b013e3181d773eb

12. Jelovac A, Landau S, Gusciute G et al (2025) Retrograde amnesia following electroconvulsive therapy for depression: propensity score analysis. BJPsych Open 11:e81. https://doi.org/10.1192/bjo.2025.25

13. Karl S, Methfessel I, Weirich S et al (2022) Electroconvulsive Therapy in Children and Adolescents in Germany-A Case Series From 3 University Hospitals. J ECT 38:249–254. https://doi.org/10.1097/YCT.0000000000000861

14. Laroy M, Emsell L, Vandenbulcke M, Bouckaert F (2025) Mapping electroconvulsive therapy induced neuroplasticity: Towards a multilevel understanding of the available clinical literature - A scoping review. Neurosci Biobehav Rev 173:106143. https://doi.org/10.1016/j.neubiorev.2025.106143

15. Methfessel I, Sartorius A, Zilles D (2018) Electroconvulsive therapy against the patients’ will: A case series. World J Biol Psychiatry 19:236–242. https://doi.org/10.1080/15622975.2017.1293296

16. Nordenskjöld A, Güney P, Nordenskjöld AM (2022) Major adverse cardiovascular events following electroconvulsive therapy in depression: A register-based nationwide Swedish cohort study with 1-year follow-up. J Affect Disord 296:298–304. https://doi.org/10.1016/j.jad.2021.09.108

17. Osler M, Rozing MP, Christensen GT et al (2018) Electroconvulsive therapy and risk of dementia in patients with affective disorders: a cohort study. Lancet Psychiatry 5:348–356. https://doi.org/10.1016/S2215-0366(18)30056-7

18. Ousdal OT, Argyelan M, Narr KL et al (2020) Brain Changes Induced by Electroconvulsive Therapy Are Broadly Distributed. Biol Psychiatry 87:451–461. https://doi.org/10.1016/j.biopsych.2019.07.010

19. Plahouras JE, Konstantinou G, Kaster TS et al (2021) Treatment Capacity and Clinical Outcomes for Patients With Schizophrenia Who Were Treated With Electroconvulsive Therapy: A Retrospective Cohort Study. Schizophr Bull 47:424–432. https://doi.org/10.1093/schbul/sbaa144

20. Rhee TG, Sint K, Olfson M et al (2021) Association of ECT With Risks of All-Cause Mortality and Suicide in Older Medicare Patients. Am J Psychiatry 178:1089–1097. https://doi.org/10.1176/appi.ajp.2021.21040351

21. Sartorius A, Demirakca T, Böhringer A et al (2019) Electroconvulsive therapy induced gray matter increase is not necessarily correlated with clinical data in depressed patients. Brain Stimul 12:335–343. https://doi.org/10.1016/j.brs.2018.11.017

22. Scalia J, Lisanby SH, Dwork AJ et al (2007) Neuropathologic examination after 91 ECT treatments in a 92-year-old woman with late-onset depression. J ECT 23:96–98. https://doi.org/10.1097/YCT.0b013e31804bb99d

23. Scholz-Hehn AD, Müller JC, Deml R et al (2019) Factors Influencing Staff’s Attitude Toward Electroconvulsive Therapy: A Comparison of New Versus Experienced Electroconvulsive Therapy Clinics. J ECT 35:106–109. https://doi.org/10.1097/YCT.0000000000000544

24. Semkovska M, Knittle H, Leahy J, Rasmussen JR (2023) Subjective cognitive complaints and subjective cognition following electroconvulsive therapy for depression: A systematic review and meta-analysis. Aust N Z J Psychiatry 57:21–33. https://doi.org/10.1177/00048674221089231

25. Semkovska M, McLoughlin DM (2010) Objective cognitive performance associated with electroconvulsive therapy for depression: a systematic review and meta-analysis. Biol Psychiatry 68:568–577. https://doi.org/10.1016/j.biopsych.2010.06.009

26. Semple DM, Suveges S, Steele JD (2024) Electroconvulsive therapy response and remission in moderate to severe depressive illness: a decade of national Scottish data. Br J Psychiatry 225:547–555. https://doi.org/10.1192/bjp.2024.126

27. Shah PJ, Glabus MF, Goodwin GM, Ebmeier KP (2002) Chronic, treatment-resistant depression and right fronto-striatal atrophy. Br J Psychiatry 180:434–440. https://doi.org/10.1192/bjp.180.5.434

28. Sigström R, Göteson A, Joas E et al (2025) Blood biomarkers of neuronal injury and astrocytic reactivity in electroconvulsive therapy. Mol Psychiatry 30:1601–1609. https://doi.org/10.1038/s41380-024-02774-4

29. Takamiya A, Sienaert P, Gergel T et al (2022) Effectiveness of electroconvulsive therapy in patients lacking decision making capacity: A systematic review and meta-analysis. Brain Stimul 15:1246–1253. https://doi.org/10.1016/j.brs.2022.09.001

30. Țăpoi C, Alexander L, de Filippis R et al (2025) Early career psychiatrists’ perceptions of and training experience in electroconvulsive therapy: A cross-sectional survey across Europe. Eur Psychiatry 67:e86. https://doi.org/10.1192/j.eurpsy.2024.1798

31. Wachtel L, Luccarelli J, Falligant JM, Smith JR (2025) Electroconvulsive therapy in autism spectrum disorders: an update to the literature. Curr Opin Psychiatry 38:79–86. https://doi.org/10.1097/YCO.0000000000000985

32. Watts BV, Peltzman T, Shiner B (2021) Mortality after electroconvulsive therapy. Br J Psychiatry 219:588–593. https://doi.org/10.1192/bjp.2021.63

33. Wells K, Hancock N, Honey A (2021) The experience of living after ECT: a qualitative meta-synthesis. J Ment Health 30:526–540. https://doi.org/10.1080/09638237.2020.1739244

34. Wheeldon TJ, Robertson C, Eagles JM, Reid IC (1999) The views and outcomes of consenting and non-consenting patients receiving ECT. Psychol Med 29:221–223. https://doi.org/10.1017/s0033291798007193

35. Zilles-Wegner D, Gather J, Hasan A et al (2025) [Access to electroconvulsive therapy for people lacking decision making capacity and as nonvoluntary treatment : Expert consensus and statement of the German Association for Psychiatry, Psychotherapy and Psychosomatics (DGPPN)]. Nervenarzt. https://doi.org/10.1007/s00115-025-01816-8

36. Beschluss des XII. Zivilsenats vom 30.6.2021 - XII ZB 191/21 -. https://juris.bundesgerichtshof.de/cgi-bin/rechtsprechung/document.py?Gericht=bgh&Art=en&nr=121501&pos=0&anz=1. Zugegriffen: 22. Juli 2025

37. Mental health, human rights and legislation: guidance and practice. https://www.who.int/publications/i/item/9789240080737. Zugegriffen: 22. Juli 2025

38. Organisationsstruktur und Tarnorganisationen. https://www.verfassungsschutz.bayern.de/weitere_aufgaben/scientology/struktur/index.html. Zugegriffen: 22. Juli 2025
